# Characteristics of immunophenotypes and immunological in tumor microenvironment and analysis of immune implication of CXCR4 in gastric cancer

**DOI:** 10.1038/s41598-022-08622-1

**Published:** 2022-04-06

**Authors:** Fang Wen, Xiaona Lu, Wenjie Huang, Xiaoxue Chen, Shuai Ruan, SuPing Gu, Peixing Gu, Ye Li, Jiatong Liu, Shenlin Liu, Peng Shu

**Affiliations:** 1 Department of Oncology, Affiliated Hospital of Nanjing University of Chinese Medicine, Nanjing, 210029 Jiangsu Province China; 2 Nanjing University of Chinese Medicine, Nanjing, 210023 Jiangsu Province China; 3 Department of Medical Oncology, Shuguang Hospital, Shanghai University of Traditional Chinese Medicine, Shanghai, China

**Keywords:** Cancer, Immunology, Gastroenterology

## Abstract

The formation of gastric cancer (GC) is a complicated process involving multiple factors and multiple steps. The tumor–immune microenvironment is essential for the growth of GC and affects the prognosis of patients. We performed multiple machine learning algorithms to identify immunophenotypes and immunological characteristics in GC patients’ information from the TCGA database and extracted immune genes relevance of the GC immune microenvironment. C-X-C motif chemokine receptor 4 (CXCR4), belongs to the C-X-C chemokine receptor family, which can promote the invasion and migration of tumor cells. CXCR4 expression is significantly correlated to metastasis and the worse prognosis. In this work, we assessed the condition of immune cells and identified the connection between CXCR4 and GC immune microenvironment, as well as the signaling pathways that mediate the immune responses involved in CXCR4. The work showed the risk scores generated by CXCR4-related immunomodulators could distinguish risk groups consisting of differential expression genes and could use for the personalized prognosis prediction. The findings suggested that CXCR4 is involved in tumor immunity of GC, and CXCR4 is considered as a potential prognostic biomarker and immunotherapy target of GC. The prognostic immune markers from CXCR4-associated immunomodulators can independently predict the overall survival of GC.

## Introduction

Gastric cancer (GC) is rated as one of the cancers with the highest morbidity and mortality globally^1^. Despite the emergence of the combined use of surgery, surveillance endoscopy, chemotherapy, radiotherapy, and various novel treatment strategies in recent years, the prognosis for patients in GC is far from optimistic due to its predisposition to invasion or metastasis and low rate of early diagnosis^2–4^. The tumor microenvironment (TME) is the environment for tumor cells to survive, which can promote tumor cell growth and metastasis and immune escape^2,3^. According to reports, changes in the content of immune and stromal cells in TME are crucial in the diagnosis and prognosis of tumors^4^. And there is a close interaction between tumor cells, stromal cells, and immune cells in the tumor immune microenvironment. These interactions promote or inhibit tumor cell growth, invasion, metastasis, and immune escape via intricate mechanisms. Immunotherapy based on immunological checkpoint inhibitors and immunotarget drugs could be a prospective replacement therapy for some cancer victims^5,6^. However, merely a few percentages of people living with cancer get help from immunotherapy. Immune escape has been proved to be one of the critical causes of the failure of immunotherapy in cancer patients^7,8^. The proportional imbalance between regulatory cells and effector cells in tumor-related microenvironment is the main pathogenesis to encourage immune escape. Some clinical trials have shown that tumor-infiltrating leukocytes are associated with clinical efficacy and cancer prognosis^9–12^. Thence, exploration of the cell types and functions of the tumor immune microenvironment is critical to grasp the tumor progression of GC and is helpful to search molecular markers with the predictive value that affect the immune response of GC patients.

Here, we investigated the characteristics of immunophenotypes and immunological in GC microenvironment, which preliminarily disclosed the involved complex immunological response processes and related regulatory mechanisms. C-X-C motif chemokine receptor 4 (CXCR4) belonged to the C-X-C chemokine receptor family is related to various cancers. The gene is a prospective therapeutic target for a variety of tumors^11–13^, including gastrointestinal cancer^14^. Subsequently, we assessed the condition of immune cells and identified the connection between CXCR4 and GC immune microenvironment, as well as the signaling pathways that mediate the immune response involved in CXCR4. Moreover, the immune predictive model was constructed by CXCR4-related immunomodulators to help the individualization therapy of cancer patients.

## Results

### Association with stromal score and immune score with survival analysis and clinical parameters

We retrieved three hundred seventy-five samples with GC and 32 normal samples from the TCGA database. The GC samples’ stromal scores and immune scores were computed by the ESTIMATE algorithm. And the extents of the score were − 1859.703 to 2072.280 and − 1056.272 to 3124.198, separately. What’s more, the ESTIMATE score was − 2471.016 to 4868.812.

**Figure 1:**
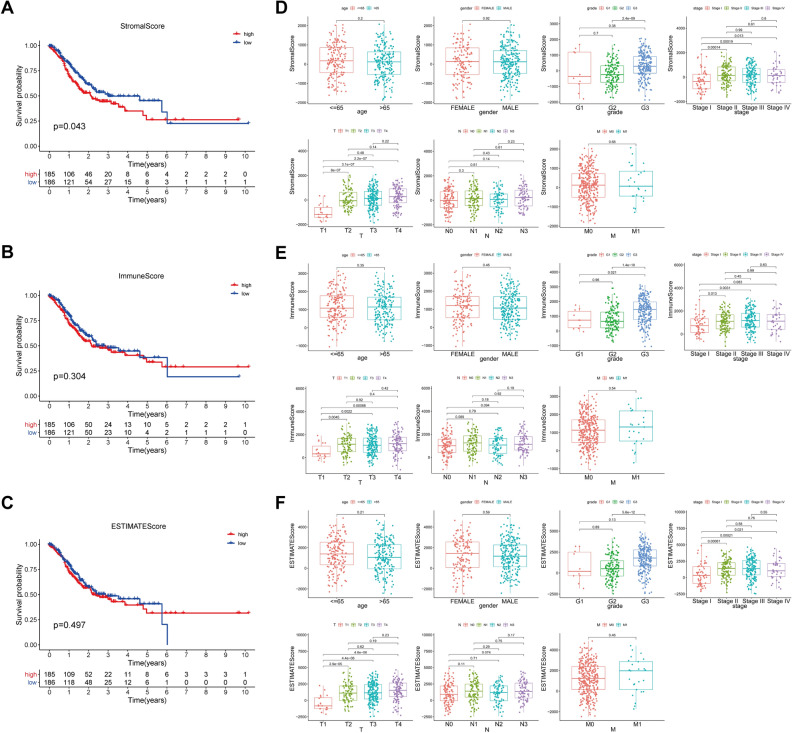
The underlying relationship between the stromal/immune/estimate scores and the overall survival and clinical parameters of the samples. (**A**) The Kaplan–Meier survival curve showed that the correlation between the high- and low-stromal score groups and 5-year survival rate. (**B**) The Kaplan–Meier survival curve showed that the correlation between the high- and low-immune score groups and 5-year survival rate. (**C**) The Kaplan–Meier survival curve showed that the correlation between the high- and low-estimate score groups and 5-year survival rate. (**D**) The box plot presented that relevance between the clinical parameters and the stromal score. (**E**) The box plot presented that relevance between the clinical parameters and the immune score. (**F**) The box plot presented that relevance between the clinical parameters and the estimate score.

To analyze the correlation between the stromal/immune/estimate scores with the OS of the GC samples, we distinguished all samples into high and low score groups. The Kaplan–Meier survival curve displayed that the low score group of the stromal score has a more extended 5-year survival period than the high score group of the stromal score (Fig. 1A). It suggested that the content of stromal cells in the GC immune microenvironment was related to the survival of patients. Survival rates were not significantly different in the high and low score groups of the immune/estimate scores (Fig. 1B,C).

By exploring the underlying connection between the stromal/immune/estimate scores with the clinical parameters, we observed that grade, stage, and tumor infiltration depth (T) had significant differences in scores (Fig. 1D-F). In terms of the median value of stage, stage II, III, IV in the stromal and estimate median scores were higher than stage I, with statistical significance. From the aspect of the median value of grade, grade 3 was higher than grade 2 in the stromal score, grade 3 was higher than grade 2 and grade1 in the immune score, and grade 3 was higher than grade 1 in the ESTIMATE score, all with statistical significance. For the median value of tumor infiltration depth (T), T2, T3, and T4 were higher than T1 in the stromal/immune/estimate scores, with statistical significance.

Taken together, the relationship investigation of stromal and immune scores with clinical parameters of GC expounded that both high stromal and immune scores were accompanied by worse tumor tissue discrepancy and serious regional penetration and had nothing to do with metastasis, suggestive of the TME have a more significant effect on primary tumor cells.

### The characteristics of the tumor microenvironment and immunophenotypes

**Figure 2:**
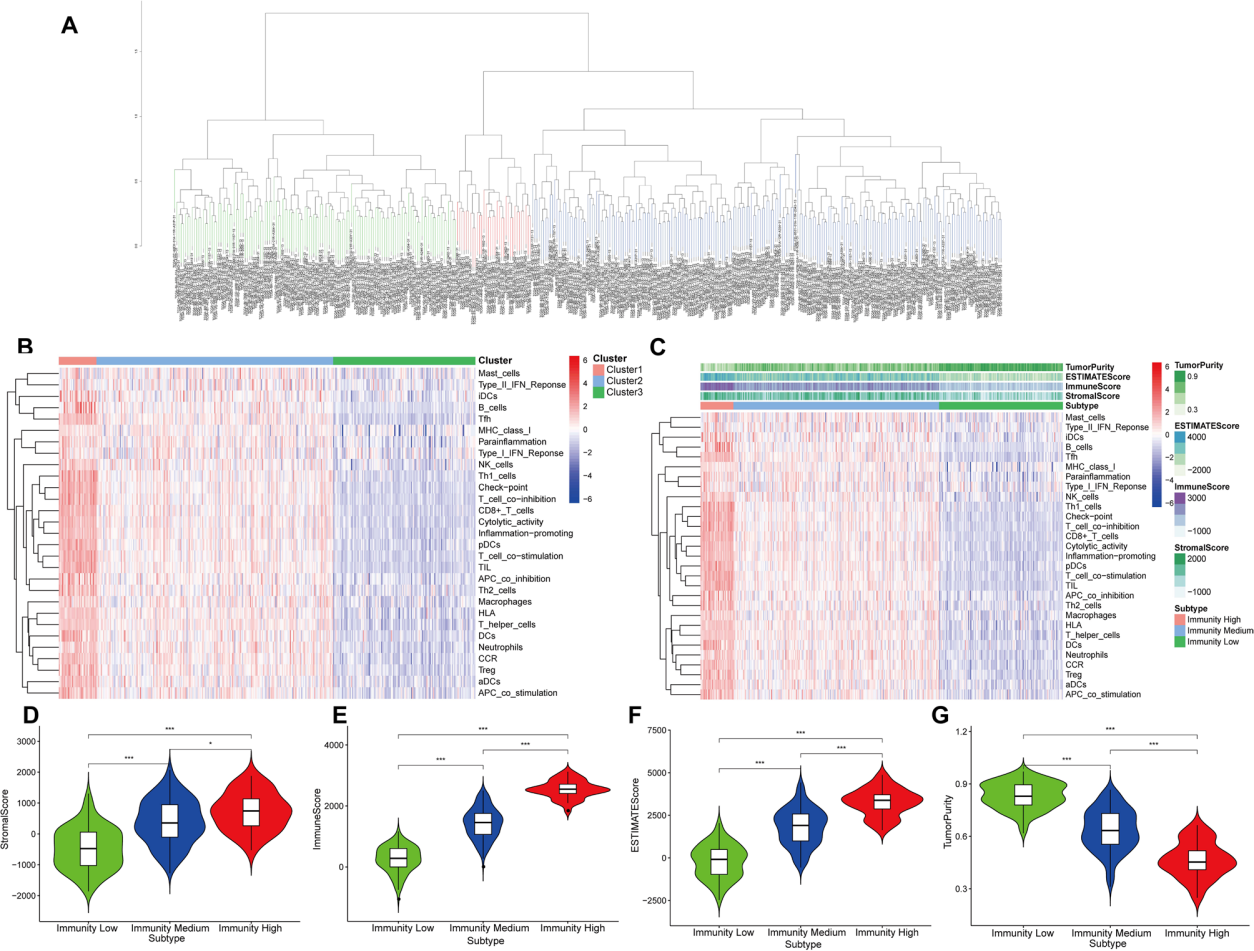
The tumor microenvironment and immunophenotypes of GC patients. (**A**) The samples were clustered into three categories by the hierarchical clustering algorithm. (**B**) The immunity characteristics of Cluster 1, Cluster 2 and Cluster 3 were compared. (**C**) Landscape of the tumor microenvironment and immunophenotypes in the High/Medium/Low Immunity groups by the tSNE algorithm and hierarchical clustering analysis. (**D**) Distribution of StromalScore in the High/Medium/Low Immunity groups. (**E**) Profile of ImmuneScore in the High/Medium/Low Immunity groups. (**F**) Profile of EstimateScore in the High/Medium/Low Immunity groups. (**G**) Profile of TumorPurity in the High/Medium/Low Immunity groups.

We combined the ssGSEA algorithm and 29 immune gene sets to evaluate the immune characteristics of GC patients. Based on the hierarchical clustering algorithm, we clustered the samples into three groups: 32 samples were clustered into cluster 1, 213 samples were clustered into cluster 2, and 128 samples were clustered into Cluster 3 (Fig. 2A). As shown in Fig. 2B, the immune feature of Cluster 1, Cluster 2, and Cluster 3 was compared, the immune characteristics of the three groups from high level to the low level were: cluster1 (High Immunity group) > cluster 2 (Medium Immunity group) > cluster 3 (Low Immunity group). It can be concluded that the High Immunity group had higher levels of immune cells and immune factors.

Next, according to the scores of the tumor microenvironment (TME) of every sample, the TME characteristics in the High/Medium/Low Immunity groups were analyzed (Fig. 2C). For the High Immunity group, StromalScore, ImmuneScore, EstimateScore, and TumorPurity were 717.300 ± 601.444, 2543.920 ± 287.686, 3261.219 ± 784.388, and 0.465 ± 0.101, separately. The Low Immunity group had the opposite trend: StromalScore, ImmuneScore, EstimateScore, and TumorPurity were − 478.587 ± 674.297, 251.226 ± 426.450, − 227.361 ± 969.231, and 0.833 ± 0.076, separately. The Medium Immunity group was somewhere between High Immunity group and Low Immunity group: StromalScore, ImmuneScore, EstimateScore, and TumorPurity were 388.558 ± 722.886, 1425.995 ± 500.151, 1814.553 ± 1072.298, 0.636 ± 0.119, separately.

In summary, the High Immunity group had the highest Stromalscore and ImmuneScore and the lowest Tumor Purity.

The characteristics of the TME in the three groups were statistically significant differences (Fig. 2D–G). In terms of tumor purity, the Low Immunity group had the highest level, while the High Immunity group had the lowest (Fig. 2G). Correspondingly, the High Immunity group had the highest infiltration degree of stromal cells and immune cells, and the lowest infiltration degree of stromal cells and immune cells in the Low Immunity group (Fig. 2D,E). As for the ESTMATE score, the High Immunity group had the highest score (Fig. 2F). All in all, with the decrease of tumor cell activity, the immune cell activity increased.

### Identification and functional enrichment analysis of DEGs between high- and low-score groups

**Figure 3:**
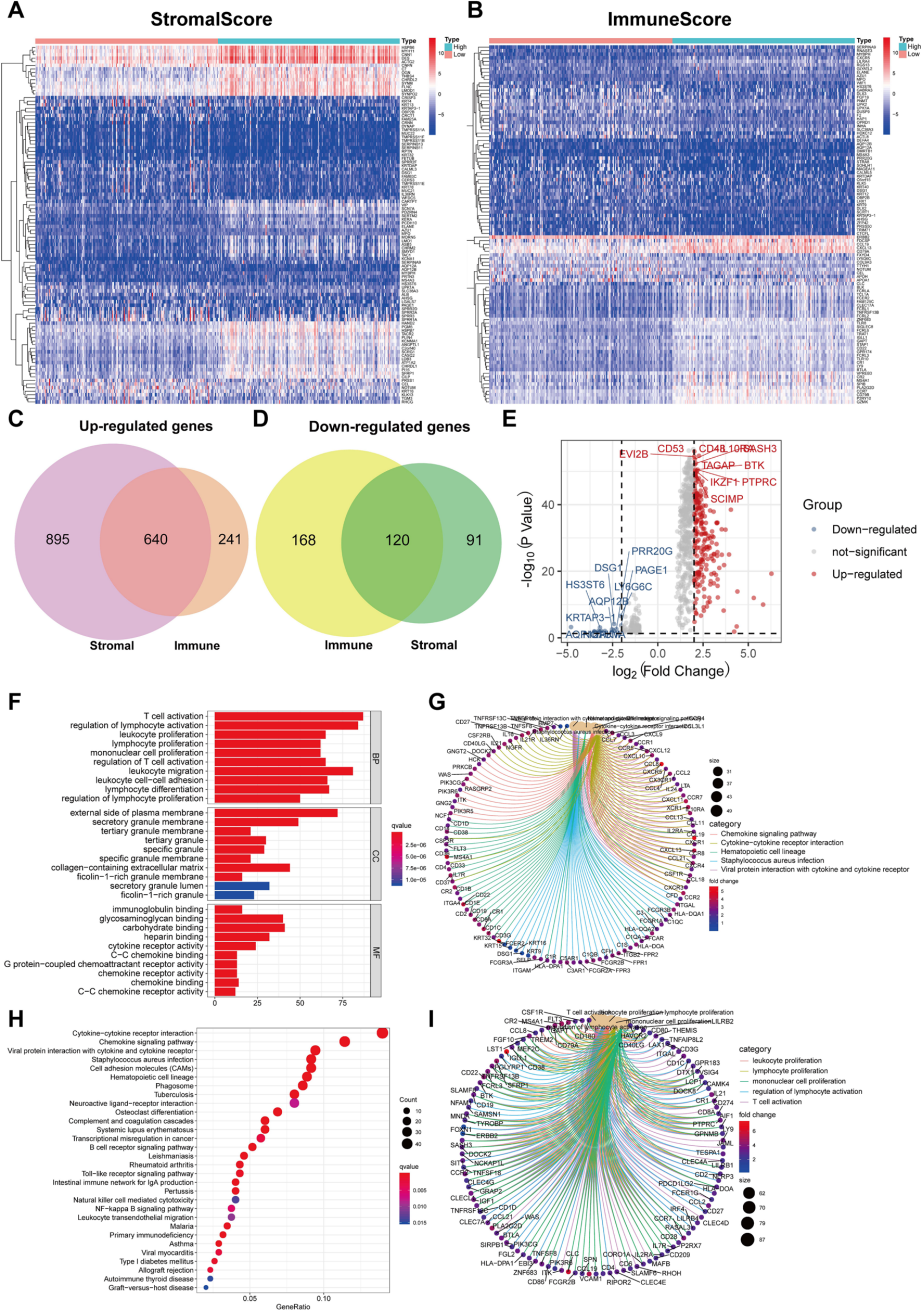
Identification and functional enrichment analysis of DEDs in GC patients. (**A**) The heat map of the DEDs with stromal scores of high score group and low score group (|logFC > 1|, FDR < 0.05). (**B**) The heat map of the DEDs with immune scores of high score group and low score group (|logFC > 1|, FDR < 0.05). (**C**) Venn diagram revealed the identical up-regulated DEDs between the stromal and immune cell groups. (**D**) Venn diagram revealed the identical down-regulated DEDs between the stromal and immune cell groups. (**E**) The volcano plot showed the top 10 up-regulated genes and the top 10 down-regulated genes. (**F**) Top 10 GO terms from functional enrichment analysis of DEGs. (**G**) The DEGs on the top 5 GO terms. (**H**) Top 30 KEGG pathway analysis of DEGs. (**I**) The DEGs on the top 5 KEGG pathway. (KEGG: www.kegg.jpkegg/kegg1.html).

To disclose the potential connection between the stromal/immune scores and the genes’ expression level of the GC samples, the differential analysis of transcriptome data of GC patients was explored (Fig. 3A,B). From the Venn diagram, there were 640 genes were up-regulated and 120 genes were down-regulated (Fig. 3C,D). The volcano plot revealed the top 10 up-regulated genes and down-regulated genes, separately (Fig. 3E).

We applied enrichment analysis on the obtained 760 DEDs (640 up-regulated genes and 120 down-regulated genes). Gene Ontology (GO) functions were mainly enriched in leukocyte proliferation, lymphocyte proliferation, mononuclear cell proliferation, regulation of lymphocyte activation, T cell activation, and lymphocyte differentiation (Fig. 3F,G), while Kyoto Encyclopedia of Genes and Genomes (KEGG) pathways (http://www.genome.jp/kegg/) were mainly enriched in viral protein interaction with cytokine and cytokine receptor, staphylococcus aureus infection, hematopoietic cell lineage, chemokine signaling pathway, cytokine-cytokine receptor interaction, cell adhesion molecules (CAMs), and phagosome (Fig. 3H,I). These consequences signify DEGs are associated with immune function.

### Identification and enrichment analysis of the PRHG

**Figure 4:**
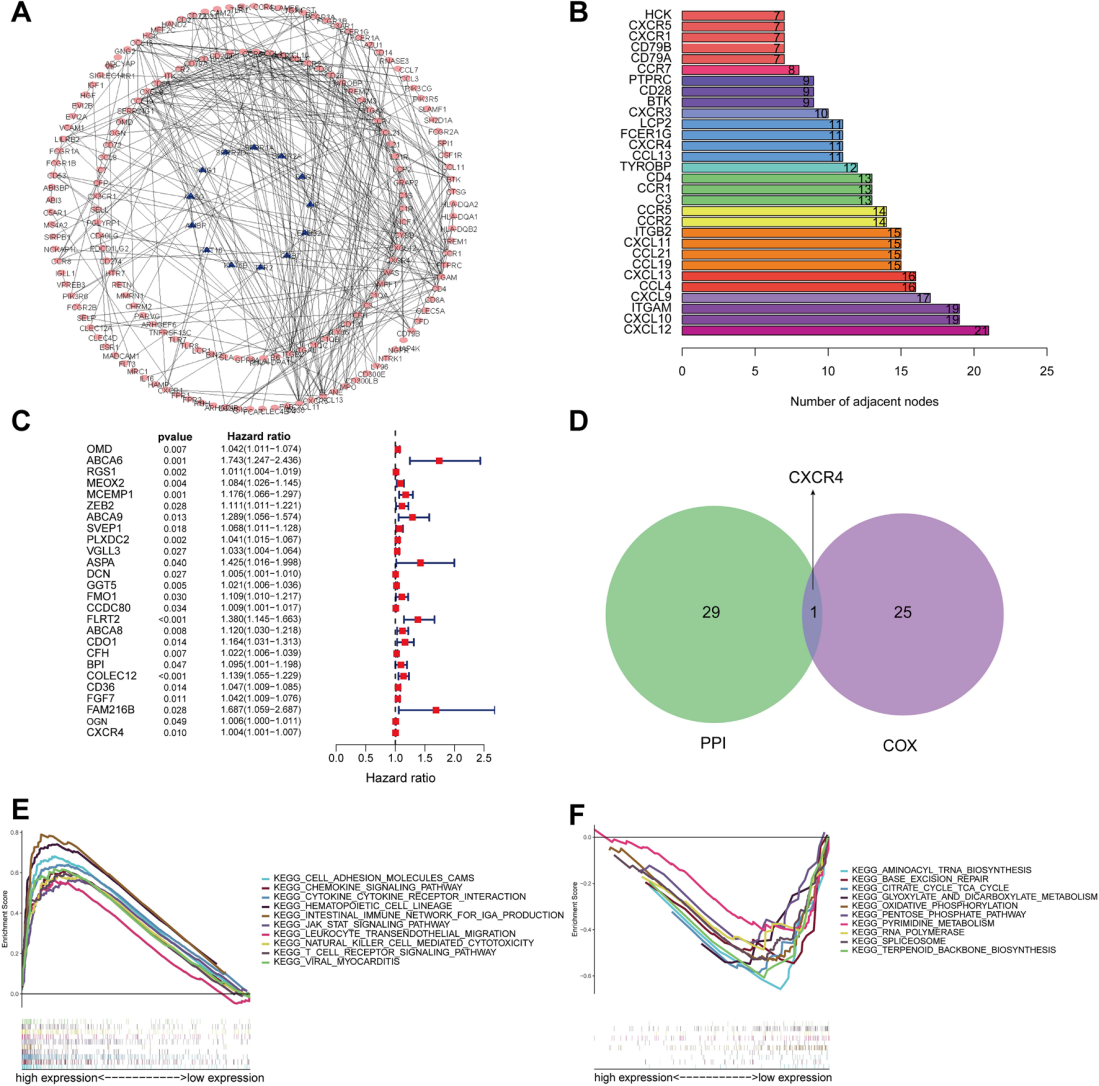
Identification and enrichment analysis of the PRHG. (**A**) The PPI network of genes with prognostic value. (**B**) The top 30 hub genes extracted from the PPI network. (**C**) The univariate COX analysis of 760 DEGs. (**D**) Identification of the hub prognostic gene. (**E**,**F**) Dissection of PRHG-associated KEGG pathways employing Gene Set Enrichment Analysis. (KEGG: www.kegg.jpkegg/kegg1.html).

We constructed a PPI network of genes with prognostication effects using the STRING network to elucidate the interrelationship between genes with prognostic values. In this PPI network, there were 188 nodes and 372 edges, with red nodes representing up-regulated genes and purple nodes representing down-regulated genes (Fig. 4A). The hub genes in the PPI network were determined by analyzing the count of neighbor nodes of each gene. Figure 4B displayed the top 30 hub genes from the PPI network. To obtain the prognostic-related genes, we carried out a univariate COX analysis of 760 DEGs (Fig. 4C). The result showed that the 26 genes related to prognosis were high-risk genes (hazard ratio, HR > 1), unfortunately, indicating a poor prognosis.

CXCR4 was the hub prognostic gene obtained by the intersection of PPI hub genes and prognostic-related genes (Fig. 4D). In vitro functional studies have shown that CXCR4 promotes the proliferation, migration and invasion of gastric cancer cells and can be used as an independent prognostic indicator for GC patients^15^. Next, CXCR4-related KEGG pathways^16^ were analyzed by GSEA software. GSEA analysis illustrated that the CXCR4 gene was relevance of some immune-related signaling pathways, involving in intestinal immune network for IgA production (NES = 2.119, *P* < 0.001), leukocyte transendothelial migration (NES = 2.188, *P* < 0.001), natural killer cell-mediated cytotoxicity (NES = 2.120, *P* < 0.001), and T cell receptor signaling pathway (NES = 2.113, *P* < 0.001) (Fig. 4E,F).

### Correlation between CXCR4 and tumor immune cell infiltration

**Figure 5:**
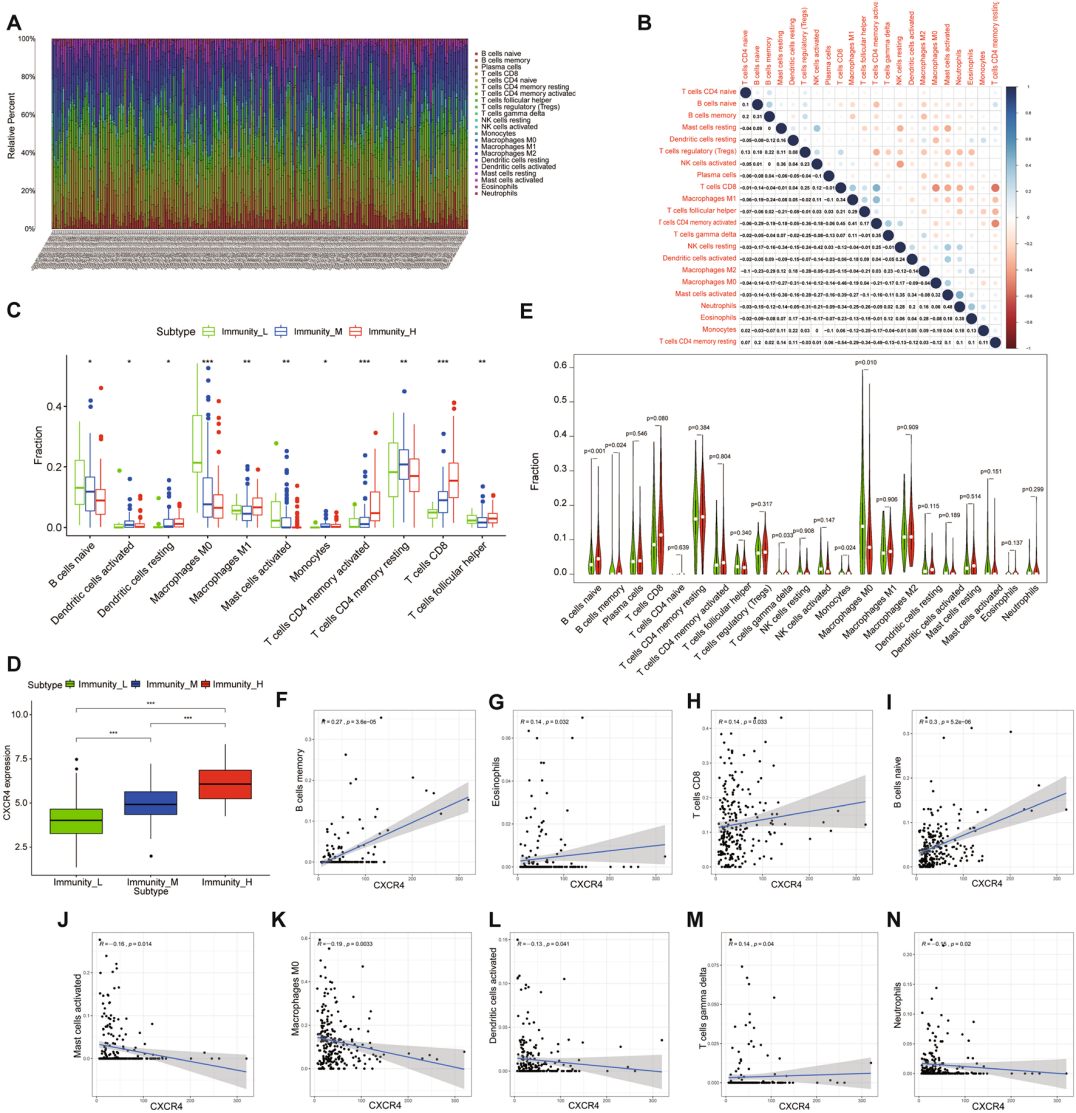
Relationship between CXCR4 and tumor immune cell infiltration. (**A**) Landscape of immune cell infiltration in TCGA-STAD samples determined by the CIBERSORT algorithm. (**B**) Various relevance forms among 26 immune cell subsets in TCGA-STAD cohorts. (**C**)The content of immune cells was significantly different among different immunity groups. (**D**)The boxplot showed that the level of CXCR4 expression was positively relevance of the infiltration degree of immune cells. (**E**) Violin plots showed the distinctions in the immune cell content between high expression group (red) and low expression group (green) of CXCR4. (**F**-**N**) Correlation analysis of CXCR4 and immune cell infiltration.

The CIBERSORT algorithm was applied to investigate the infiltration degree of immune cells in GC samples (Fig. 5A). Furthermore, various relevant forms among the immune cells were revealed in TCGA cohorts (Fig. 5B). As a result, T cells CD4 memory activated was negatively correlated with B cells naïve, T cells regulatory (Tregs), and T cells CD4+ (CD4+ T) memory resting, but positively relevant to Macrophages M1 and T cells CD8+ (CD8+ T). Also, the contents of immune cells varied among different immune groups (Fig. 5C). In detail, Dendritic cells resting, Macrophages M1, T cells CD4 memory activated, T cells CD8, and T cells follicular helper had the highest fraction in the High Immunity group. Dendritic cells activated, Monocytes, and T cells CD4 memory resting had the highest fraction in Medium Immunity group. B cells naïve, Macrophages M0 and T Mast cells activated had the highest fraction in the Low Immunity group.

The expression level of CXCR4 was positively relevant to the infiltration degree of immune cells (Fig. 5D). By analyzing the differences of 22 immune cells in the high expression group and low expression group of CXCR4, we discovered CXCR4 gene expression was significantly correlated with B cells naïve, B cells memory, T cells gamma delta, Monocytes, and Macrophages M0 (Fig. 5E). As shown in Fig. 5F-N, B cells naïve, B cells memory, CD8+ T, T cells gamma delta, Mast cells activated, and Eosinophils infiltration were positively relevant to the high expression group of CXCR4, while Neutrophils, Macrophages M0, Mast cells activated, and Dendritic cells (DCs) activated infiltration reached an inverse outcome.

### The prognostic implication of CXCR4-related immunomodulators in GC

**Figure 6:**
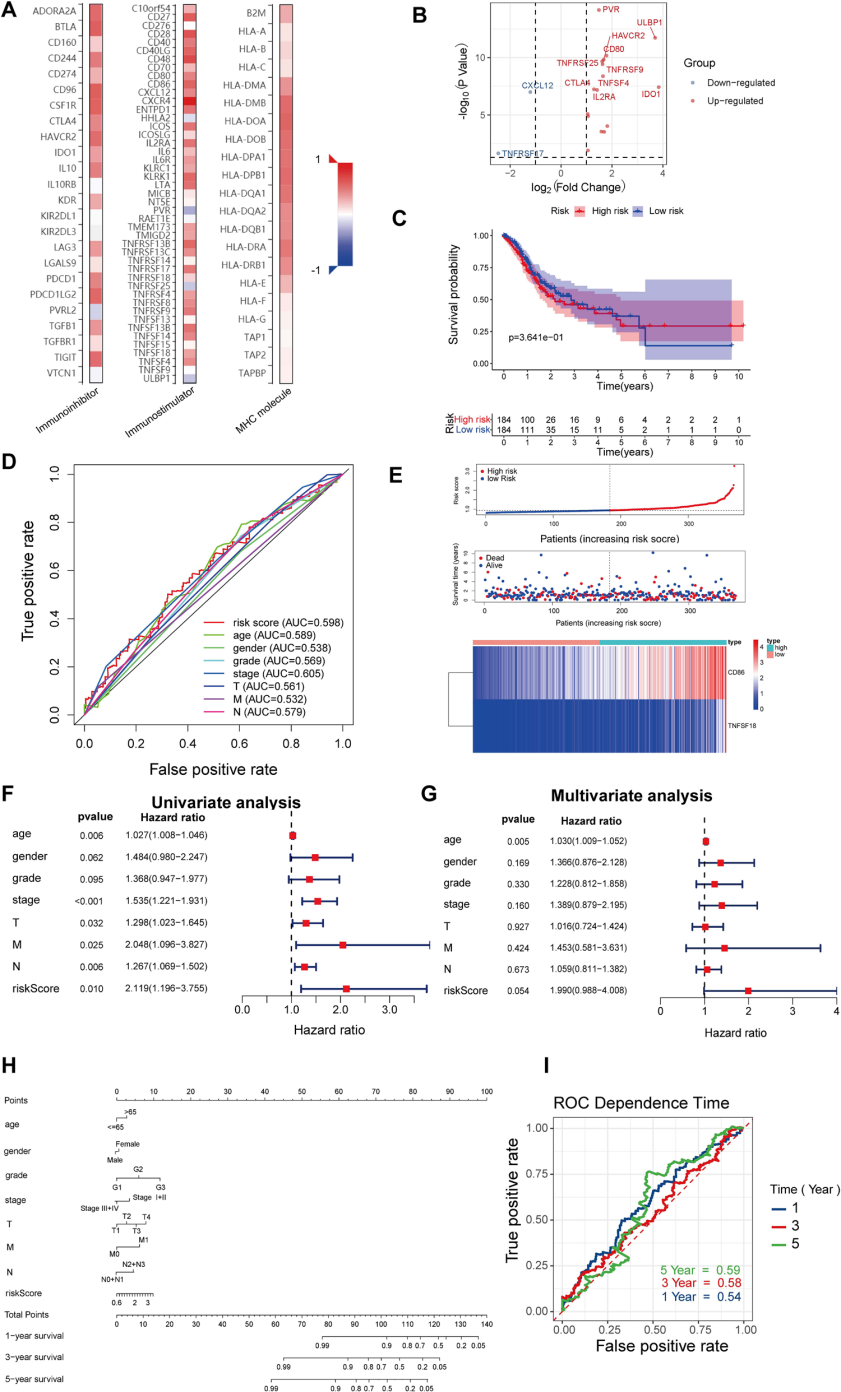
Identification and prognostic value of immunomodulators associated with the CXCR4 in GC. (**A**) The heatmaps of relevance between the immunomodulators and the CXCR4 in GC. (**B**) The volcano plot shows the genes that were connected with these immunomodulators. Red signifies the top 10 up-regulated genes; blue signifies the top 10 down-regulated genes. (**C**) Kaplan–Meier curves for GC regarding the risk scores. (**D**) ROC curves of the risk score and other clinical indexes. (**E**) Distribution of risk scores, and survival statutes, as well as gene expression profiles of GC. (**F**) Univariate Cox regression analyses of the risk score in GC regarding overall survival. (**G**) Multivariate Cox regression analyses of the risk score in GC regarding overall survival. (**H**) Nomogram created along with the risk genes and clinical index. (**I**) Time-dependent ROC curve of the nomogram exhibits the ROC curve and AUC for 1-, 3-, and 5-year survival, separately.

CXCR4-related immunomodulators in GC were obtained through the TISIDB database. We identified 20 immunoinhibitors (ADORA2A,BTLA,CD160,CD244,CD274,CD96,CSFIR,CTLA4,HAVCR2,IDO1,IL10,KDR,LAG3,LGALS9,PDCD1,PDCD1LG2,PVRL2,TGFB1,TGFBR1, and TIGIT), 37 immunostimulators (CXCR4,ENTPDI,HHLA2,ICOS,ICOSLG,IL2RA,IL6,lL6R,KLRCI,KLRKI,LTA,PVR,TMEM173,TMlGD2,TNFRSF13B,TNFRSF13C,TNFRSF17,TNFRSF18,TNFRSF25,TNFRSF4,TNFRSF8,TNFRSF9,TNFSF13B,TNFSF14,TNFSF18,TNFSF4, and ULBP1) and 16 MHC molecules(MHC molecule: B2M,HLA-A,HLA-B,HLA-C,HLA-DMA,HLA-DMB,HLA-DOA,HLA-DOB,HLA-DPA1,HLA-DPB1,HLA-DQAI,HLA-DQA2,HLA-DQBI,HLA-DRA,HLA-DRB1, and HLA-E) significantly associated with CXCR4 in GC (Fig. 6A). The volcano plot showed the 10 top genes that were closely related to these immunomodulators (Fig. 6B).

To probe the predictive effects of CXCR4-related immunomodulators in GC, we performed these molecules into univariate and multivariate Cox regression analysis. These analyses resulted in an optimal 2-gene prognostic model of GC. And we constructed a 2-gene prognostic model. The Cox regression analysis and the biological functions of the genes are presented in Table 1. The risk scores were computed through a specific formula. The Kaplan–Meier survival curve explained that the low-risk scores patients had a higher 5-year survival rate than the high-risk scores patients (*P* < 0.001) (Fig. 6C). The risk score’s area under the curve (AUC) value was 0.598 (Fig. 6D). The distribution of risk scores, survival statuses, and the expression levels of risk genes for GC was visualized in Fig. 6E. The results represented the distribution of patients via ranking the risk score. The dot chart displayed that most patients in the low-risk group were alive status. And, the heatmap revealed the high expression of CD86 in the high-risk group and low expression in the low-risk group, while the expression of TNFSF18 was reversed. Furthermore, we analyzed the correlation between the risk score and the survival rate in TCGA-STAD. Univariate Cox regression analysis represented that the variables of age, stage, T, M, N, and risk score were significantly correlated with the OS (*p* < 0.05) (Fig. 6F). Multivariate Cox regression analysis indicated that the variables of age score were independently correlated with the OS (*p* < 0.05) (Fig. 6G). Moreover, univariate Cox regression disclosed that the risk score was an independent predictor of GC independent of age, gender, stage, T, M, and N (HR = 2.119, 95% CI = 1.196–3.755, *P* = 0.010).

**Table 1:** Multivariate Cox regression analysis of immune infiltration cells in GC.

Immune cells	Coef	HR	Lower 95% CI	Upper 95% CI	*p* value	Sig
B cell	− 1.872	0.154	0.000	1324.554	0.686	–
CD8 T cell	− 4.799	0.008	0.000	0.961	0.048	*
CD4 T cell	− 5.688	0.003	0.000	7.542	0.148	–
Macrophage	1.979	7.236	0.006	8707.998	0.584	–
Neutrophil	− 2.622	0.073	0.000	631.194	0.571	–
Dendritic	3.699	40.399	0.372	4388.085	0.122	–

R square = 0.036 (max possible = 9.04e−01); Likelihood ratio test *p* = 8.55e−02; Wald test *p* = 2e−01; Score (log-rank) test *p* = 2.09e−01.

Ultimately, based on the risk score and clinical parameters, we built a prognostic nomogram in GC to foresee the personals' survival opportunities (Fig. 6H). We also utilized time-dependent ROC to evaluate the predictive discrimination of the nomogram (Fig. 6I). The AUC values of the 1-year, 3-year, and 5-year were 0.59, 0.58 and 0.54, separately.

## Discussion

TME is a complicated environment that involves various cells and cellular molecules, which has been proven to play an essential role in the treatment or prognosis of tumors and has become a research hotspot in immunotherapy and precision treatment of malignant tumors^17,18^. Our results that analyzed by the ESTIMATE algorithm revealed that the low stromal score group had a higher 5-year survival period than the high stromal score group, supporting the conclusions that the content of stromal cells in the GC immune microenvironment was related to the survival of patients, which was similar to the study of Zhou’s team^19^. By analyzing the relationship between stromal scores, immune scores and clinical parameters of GC, we found that the patients with high stromal and immune scores had a low degree of tumor differentiation and severe local invasion, but had little effect on metastasis. Based on the hierarchical clustering algorithm, the patients were clustered into immune groups of different degrees. The findings suggested the High Immunity group had the highest infiltration degree of stromal cells and immune cells, and the lowest level of tumor purity. This result was similar to previous studies, that was, patients with abundant stromal cells and immune cells in TME had a higher immunological response^19^.The functional enrichment analysis of 760 DEGs showed that the DEGs were associated with immune function. In our study, all of the 26 genes related to prognosis were high-risk genes in GC, unfortunately, indicating poor prognosis.

The intersection of 30 PPI hub genes and 26 prognostic-related genes was taken to obtain the CXCR4 gene. CXCR4 can promote the growth, invasiveness, and metastatic spread of tumor cells ^20,21^. The expression of CXCR4 in various cancers was firmly related to metastasis and worse OS, providing a basis for CXCR4 targeted therapy^22–25^, building a therapeutic rationale for CXCR4 targeting. Experiments confirmed that CXCR4 was related to the pathogenesis or prognosis of GC and other malignant tumors ^14,26,27^. Generally, CXCR4’s high expression in cancers is related to a poor prognosis. Professor Xue's research confirmed that patients with CXCR4 low expression had a better prognosis than patients with high expression^15^. CXCR4-related KEGG pathways included intestinal immune network for IgA production, leukocyte transendothelial migration, natural killer cell-mediated, and T cell receptor signaling pathway. CIBERSORT algorithm results illustrated that the high expression group of CXCR4 was positively related to B cells naïve, B cells memory, CD8+ T, T cells gamma delta, Mast cells activated, and Eosinophils infiltration, while Neutrophils, Macrophages M0, Mast cells activated, and Dendritic cells (DCs) activated infiltration received the opposite results. CD8+ T has the anti-tumor immune function and accelerates apoptosis of tumor cells, and has favorable treatment effects on numerous cancers, including GC^28–30^. B cells have a dual impact on promoting immune responses and anti-tumor immune responses ^31,32^, and are related to the favorable prognosis for several cancer types ^33,34^. Tumor-associated macrophages (TAMs) motivate tumor angiogenesis, maintain stem cells, and suppress immune responses ^35^. The high infiltration of TAM promotes tumorigenesis and is related to poor OS in many tumors^36^. DCs, a member of the APCs, can promote naive T cell activation and participate in the immune memory response processes in tumors ^37,38^.

In vitro experiments demonstrated that tyrosine kinase inhibitors could pertinently enhance the expression of CXCR4 on Natural killer (NK) cells and monocytes^39^. Preclinical trials confirmed the drugs that anti-CXCR4 inhibitors had anti-tumor activity against HER2 subtype of breast cancer^40^. According to report, anti-CXCR4 inhibitors could not only inhibit CXCR4 signaling and mobilize T cells of pancreatic cancer in vivo, but also illustrated higher anti-cancer effect if along with anti-PD-L1 drugs^41–43^, exhibiting similar promising preclinical results. It has been found that the introduction of oncolytic viruses equipped with CXCR4 antagonists can restore pathological signaling, diminish metastasis and decrease regulatory T cell enrichment in ovarian cancer ^44^. Moreover, NK cells co-expressed with the CXCR4 increased the infiltration of NK cells and enhanced the killing effect of tumor cells ^45^. Especially, studies have found that the CXCR4-CXCL12 axis mediated the development of B, T, and NK cells ^46,47^. Besides, CXCL12-KDEL retention protein was used to block colon cancer cells, resulting in repression of the signaling involved in CXCR4 and a significant reduction in the metastatic cancer growth^48^. Also, the CXCR4-CXCL12 axis inhibition could profit the patients of GC^49^.

In the work, we identified that the CXCR4 is firmly connected with GC immune microenvironment. The expression of CXCR4 was the relevance of immunomodulators and the infiltration levels of immune cells. We established an immune-gene model for GC using CXCR4-associated immunomodulators. The risk scores based on the gene signatures were closely related to prognosis in GC. Subsequently, the nomogram was established for the use of personalized prognosis prediction. And, we performed time-dependent ROC to assess the precise discrimination of nomogram. The results of this study revealed the risk scores based on CXCR4-related immunomodulators could distinguish risk groups composed of differentially expressed immune genes. Our work might promote the development of verification signatures for the prognosis of GC.

In summary, our results suggest that CXCR4 may also play a role in the regulation of GC immune microenvironment. The prognostic immune markers based on CXCR4-related immunomodulators can Individually predict the OS of GC. Prospective studies need to perform to validate the clinical application of biomarkers in individualized treatment of GC.

## Methods

### Data collection

The patients’ information was received via The Cancer Genome Atlas (TCGA, https://portal.gdc.cancer.gov/) database. The collation and extraction of data information used Perl scripts.

### Survival analysis and related clinical parameters analysis of the tumor microenvironment

The R package "ESTIMATE" was used to score stromal and immune cells in TME. Use R packages "survival" and "survminer" to combine the TME scores (Stromal score, Immune score, ESTIMATE score) with the survival data of the samples and analyze them. These samples with different immune activities were clustered according to immune scores. In conformity with the median value of the TME scores, the samples were divided into high- and low-score groups, and the survival differences between the two groups were explored. The R package "ggpubr" was applied to clarify the difference between TME scores and clinical parameters and different immune activity groups.

### Screening of differentially expressed genes and the hub genes

The differentially expressed genes (DEGs) between high- and low- score groups of the stromal score and immune score were analyzed, separately. The filter condition was |log2 (fold change, FC) > 1| and false discovery rate (FDR) < 0.05. The DEGs in stromal cells were intersected with those in immune cells, and the intersecting DEGs were enriched and investigated. The network by protein–protein interaction (PPI) of the intersecting DEGs was built via the STRING database (https://string-db.org/) and Cytoscape software version 3.7.1(https://cytoscape.org/). The count of neighbor nodes of every gene in PPI was analyzed and the hub gene was obtained. Univariate Cox regression analysis was performed to select the genes connected with prognosis. The prognosis-related hub gene (PRHG) was obtained by the intersection of the prognosis-related genes and hub genes.

### Enrichment analysis of the prognostic-related hub genes

Gene Set Enrichment Analysis (GSEA) v4.0.1 software (https://www.gsea-msigdb.org/gsea/login.jsp) was performed to disclose PRHG related pathogenesis and biological signaling pathways.

### The correlation analysis between PRHG and immune cells in TME

The relative content of immune cells in each sample was calculated by the CIBERSORT algorithm (R Script v1.03), and the relationship between different immune cells was determined by R package "corrplot". Next, we analyzed the correlation between PRHG expression and immune cell infiltration in GC. CIBERSORT R script obtained from the CIBERSORT website (https://cibersort.stanford.edu/) was explored to examine the relationship between the hub gene and immune grouping and different immune cells.

### Construction of the immune prognosis model based on PRHG-related immunomodulators

We searched the TISIDB database (http://cis.hku.hk/TISIDB/) for immunomodulators related to PRHG, including immunoinhibitor, immunostimulator, and major histocompatibility complex (MHC) molecule (Spearman correlation test, *P* < 0.05).

A prognostic multiple immune-gene model was constructed based on PRHG-related immunomodulators. The possible immune genes were initially identified through univariate Cox analysis. The expression values of immune genes were weighted by the regression coefficient of the Cox regression model to calculate the risk score of every patient. ${\text{Risk}}\;{\text{core = }}\sum\nolimits_{{{\text{i = }}1}}^{{\text{n}}} {{\text{coefi}}\;{\text{X}}\;{\text{id}}}$. Kaplan–Meier survival curve was performed to evaluate the relation between the immune prognosis model and OS.

### Independence of the immune prognosis model

Univariate and multivariate Cox regression analysis were utilized to detect the GC patients’ prognosis. The time-dependent receiver operating characteristic (ROC) curves were utilized to study the prediction value of the risk score via the "SurvivalROC"^50^ of R package. Furthermore, Nomogram^51^ was constructed to predict the survival of GC patients at 1 year, 3 years, and 5 years.

### Statistical analysis

R software (version 3.6.1, https://cran.r-project.org/bin/windows/base/) was applied to perform all statistical analyses. Wilcoxon test was suitable for comparison of data between two groups and Kruskal–Wallis test was performed for three or more groups. Kaplan–Meier curves and log-rank tests explored the survival data. Qualitative variables were compared using Pearson χ^2^ test or Fisher's exact test. Spearman correlation test was performed to analyze the correlation between immune cells. For all comparisons in this study, *p* value < 0.05 was believed to be reliable.

### Ethics approval and consent to participate

Not applicable.

### Consent for publication

Consent for publication in this magazine.

## Data Availability

All data and materials are available. The data that support the findings of this study are available from The Cancer Genome Atlas (TCGA, https://portal.gdc.cancer.gov/) database.
